# The Potential of an *in Vitro* Digestion Method for Predicting Glycemic Response of Foods and Meals

**DOI:** 10.3390/nu8040209

**Published:** 2016-04-08

**Authors:** Konstantina Argyri, Adelais Athanasatou, Maria Bouga, Maria Kapsokefalou

**Affiliations:** Unit of Human Nutrition, Department of Food Science and Human Nutrition, Agricultural University of Athens, 75 Iera Odos Str., Athens 11855, Greece; nargiri@gmail.com (K.A.); dathanasatou@gmail.com (A.A.); mairabouga@gmail.com (M.B.)

**Keywords:** glycemic index, glycemic load, glycemic response

## Abstract

Increased interest in glycemic response derives from its linkage with chronic diseases, including obesity and type 2 diabetes. Our objective was to develop an *in vitro* method that predicts glycemic response. We proposed a simulated gastrointestinal digestion protocol that uses the concentration of dialyzable glucose (glucose in the soluble low molecular weight fraction of digests) as an index for the prediction of glycemic response. For protocol evaluation, dialyzable glucose from 30 foods or meals digested *in vitro* were compared with published values for their glycemic index (GI) (nine foods), glycemic load (GL) (16 foods) and glycemic response (14 meals). The correlations were significant when comparing dialyzable glucose with GL (Spearman’s rho = 0.953, *p* < 0.001), GI (Spearman’s rho = 0.800, *p* = 0.010) and glycemic response (Spearman’s rho = 0.736, *p* = 0.003). These results demonstrate that despite limitations associated with *in vitro* approaches, the proposed protocol may be a useful tool for predicting glycemic response of foods or meals.

## 1. Introduction

Diets with high glycemic response, which cause a high increase in blood glucose, have been associated with increased risk of chronic diseases, including obesity, type II diabetes, cardiovascular disease and certain types of cancer [1,2,3,4,5,6,7]. Therefore, in some cases, glycemic response has been employed as a criterion to guide the selection of foods in balanced dietary schemes or in the formulation of new products [8].

To allow the evaluation of glycemic response, glycemic index (GI) [9] and glycemic load (GL) have been introduced [10]. GI, defined as the incremental area under the blood glucose response curve of a 50 g carbohydrate portion of a test food, is expressed as a percentage of the response to the same amount of carbohydrate from a standard food taken by the same subject [8]. GL, which takes into account how much carbohydrate a serving of a food contains, may be calculated by multiplying the GI of a food by the amount of available carbohydrate in the portion of food consumed [4]. GI and GL are measured within human feeding studies, and thus these have been considered to provide the only valid estimates of glycemic response in nutritional epidemiology [11]. However, the *in vivo* measurement of glycemic response requires the recruitment of volunteers under ethical committee approval, the availability of medical personnel for blood drawing, and disposal procedures for clinical waste; therefore, at least for some experiments that screen a large number of foods and meals, or laboratories that are not set up to conduct clinical studies, *in vivo* measurements may be regarded as complicated, expensive and time consuming [12]. Therefore, there is a need for reliable, simple, and inexpensive *in vitro* tools which, although will not substitute the measurement of GI or GL, will be a suitable indicator of these value, and may act as a precursive screening method to *in vivo* measurements.

Various *in vitro* methods have been proposed in order to predict the glycemic properties of foods [13,14,15,16,17,18,19]. *In vitro* methods measure the digestibility of carbohydrates after a procedure that mimics the oral, gastric and intestinal phases of human digestion [20], although not all studies include the oral phase [21,22]. Further differences amongst methods include the duration of each digestion phase, the type of enzymes present, and/or the indices that are employed (e.g., rapidly available glucose, hydrolysis index, and glycemic glucose equivalents) [12,13]. Although some methods are reasonably simple [13], others demand sophisticated laboratory equipment, expensive reagents, trained personnel and complex calculations [23]. Although the large variability in existing methods offers multiple tools for approaching diverse research objectives, the lack of a standardized, validated and widely accepted *in vitro* protocol is challenging. Furthermore, due to the increasing interest in the prediction of glycemic response of foods and meals, particularly within routine applications, there is still a need for the development of protocols that are simpler than the existing ones; this need emerges because of the increasing interest for the prediction of glycemic response of foods and meals particularly in routine applications. For example, some applications, such as the screening processes that take place during the development of new food products, simple methods that show trends and comparisons amongst foods may be preferable to more complex methods that predict GI through linear regression models.

The present study aims to: (i) propose an *in vitro* digestion protocol that offers a simple, rapid and inexpensive prediction of glycemic response; and (ii) evaluate its reliability through the comparison of *in vitro* results with a series of foods and meals with published values of GI, GL or glycemic response.

## 2. Materials and Methods

### 2.1. Foods Subjected to in Vitro Digestion

A series of 9 foods with published GI (Table 1) and 16 foods with published GL (Table 2) were selected from the International Table of GI and GL values [24]. The selection criteria were the variety in composition, aiming to include foods with low, medium and high glycemic response and their availability in the Greek market. Both liquid and solid foods were included in the study.

Foods were purchased from two local supermarkets (the footnotes of Table 1 and Table 2 outline the brand and respective food number from the “International Table of Glycemic Index and Glycemic Load Values” for each product [24]). The nutrient content of processed and packaged foods was recorded from the nutrient information provided by the manufacturer, and the nutrient content of fruit was derived from food composition tables [25]. All foods were prepared as described previously [24] and provided carbohydrate content was identical to that of the respective foods in the “International Table of GI and GL”. The only exception was boiled spaghetti, which had higher carbohydrate content and therefore an adjustment had to be made in the amount digested *in vitro*.

### 2.2. Meals Subjected to in Vitro Digestion

A series of meals (Table 3) was included in the experimental design and were prepared as previously described [26]. In that study, 70 diabetic subjects (42 men and 28 women, mean age; 64 years (SD: 8.7), duration of diabetes; 11.6 years (SD: 5.2)) were randomly recruited to consume the following meals: a slice of white bread with a slice of low fat cheese (10% fat) (basic meal) and either a dessert targeted to diabetics (D-dessert) or a dessert targeted to the general population (C-dessert) [26]. The nutrient content of desserts and basic meal was provided by the manufacturer [24].

### 2.3. Overview of Foods or Meals Subjected to in Vitro Digestion

Foods or meals in Table 1, Table 2 and Table 3 were prepared for the *in vitro* digestion as follows.

For foods described in Table 1, a portion that provides 0.25 g carbohydrates (1/200 of the amount of carbohydrates consumed *in vivo* [24]) was used. For foods described in Table 2, 1/100 of the amount of food consumed *in vivo* [24] was used. These portions when homogenized to 2 mL, which is the amount digested in the six-well plates, provided an adequate amount of dialyzable glucose to be detected by the proposed method; it should be noted that foods with carbohydrate content less than 15 g/100 g would require more than 1.8 g of food/2 mL homogenate in the basis of GI, and therefore could not be tested with the proposed setup; however, these foods were included in the experimental design when GL was considered because, in this case, the required amount was below the volume restriction of the setup. For this reason, some foods were selected on the basis of their GI or of their GL.

For meals described in Table 3, a portion of 1/10 of the meal consumed *in vivo* was used in order to mimic the amount of meals that was consumed *in vivo* [26].

### 2.4. In Vitro Digestion Protocol

The digestion protocol was based on procedures developed in the past for predicting glycemic response [15,27,28] or mineral bioavailability [29]. Briefly, the *in vitro* digestion proceeded in two phases.

**Figure 1:**
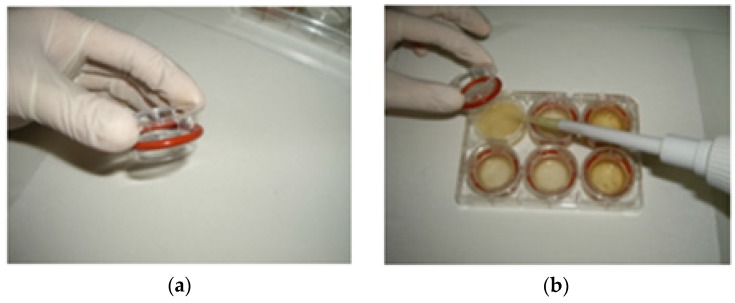
(**a**) A cylindrical insert with a piece of dialysis membrane fastened to one end with an elastic band; (**b**) The inserts are placed in 6-well plates in a way that the membrane is in contact with the digest.

In the first phase, samples were mixed with an equal weight of water, homogenized (Ultra Turrax T25 Basic, Cole-Parmer Instrument Co. Ltd., London, UK), and incubated with α-amylase (185 U/g available carbohydrate, α-amylase from human saliva, type XIII-A A1031-1KU, Sigma-Aldrich, Taufkirchen, Germany) at 37 °C for 15 min in a shaking incubator (ES-20/60, speed at 110 rpm, Biosan, Riga, Latvia), in order to mimic the oral digestion. Subsequently, the pH was adjusted to 2.5 with 0.1 M HCl. In cases that a food’s homogenate volume was less than 2 mL, water (pH adjusted to 2.5 with 0.1 M HCl) was added up to 2 mL and transferred in duplicates to wells in a six-well plate. In each well, 0.1 mL of pepsin (porcine pepsin preparation, suspended in 4 g/100 mL in 0.1 M HCl, porcine pepsin, P-7000, Sigma-Aldrich, Taufkirchen, Germany) was added and the plates were placed on a shaking incubator at 37 °C for 2 h, simulating the gastric phase of human digestion. After 2 h, a cylindrical insert, with a piece of dialysis membrane (molecular weight cutoff of 6–8 kDa, Spectrum Laboratories, Inc., Rancho Dominguez, CA, USA) fastened to one end with an elastic band (Figure 1a) was placed in each well in such a way that the membrane was in contact with the digest (Figure 1b). Each ring was filled with 2 mL 0.1 M PIPES buffer pH 6.5 (piperazine-1,4-bis (2-ethane-sulfonic acid) disodium salt (P3768, Sigma-Aldrich, Taufkirchen, Germany)), simulating the gradual increase of pH in the human small intestine. The plates were incubated for another 30 min, shaking at 37 °C.

The second phase of the *in vitro* digestion started after the end of this incubation period and lasted 120 min. An aliquot (0.2 mL) from the dialysate was taken (*t* = 0 min). Subsequently, the insert was carefully removed and 10 μL of amyloglucosidase (3260 U/mL amyloglucosidase from *Aspergillus niger* E-AMGDF, Megazyme Inc., Chicago, IL, USA) and 0.5 mL of a pancreatin–bile salt mixture (0.2 g porcine pancreatin from porcine pancreas, P-1750 Sigma, and 1.2 g bile extract, B-8631 Sigma, suspended in 100 mL 0.1 M NaHCO_3_) was added on to each digested sample. The cylindrical insert was placed back and the incubation continued in a shaking incubator for 2 h, taking aliquots (0.2 mL) every 30 min from the dialysate for the determination of glucose (*t* = 30 min, *t* = 60 min, *t* = 90 min, *t* = 120 min, where *t* = 0 min is set at the start of the second phase of the *in vitro* digestion procedure). The digested samples (0.2 mL aliquots) were mixed immediately with 0.8 mL ethanol in a microcentrifuge tube and 30 min later the tubes were centrifuged for 10 min at 5000 rpm at 20 °C (Mikro 200R, Hettich, Tuttlingen, Germany) to clarify the ethanol supernatant fraction before analysis of sugars. Dialyzable glucose, *i.e.*, the concentration of glucose in the soluble and low molecular weight fraction of the digest, was tested as an index for the prediction of glycemic response.

**Figure 2:**
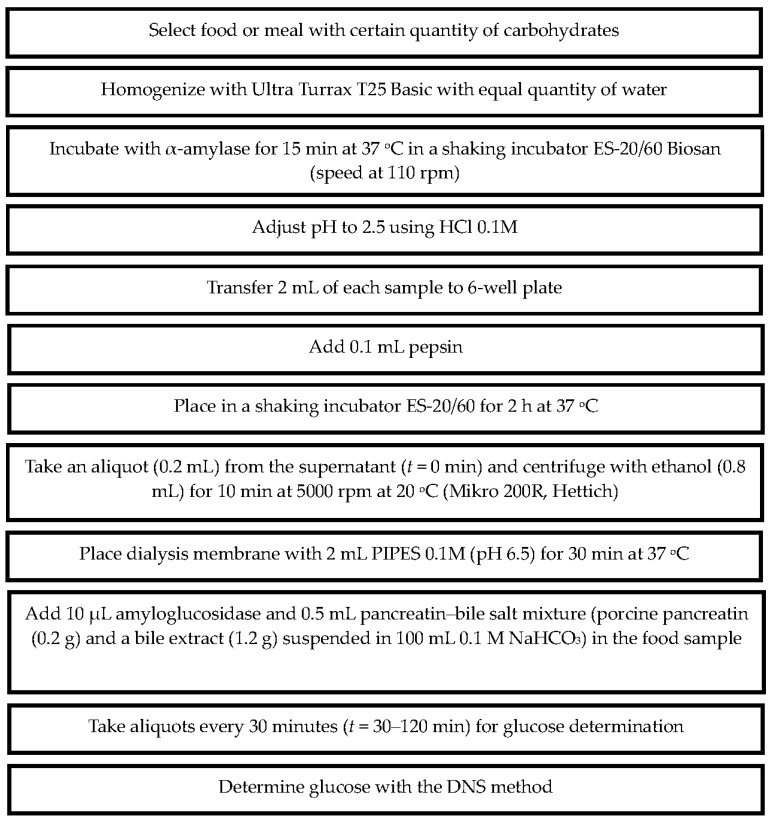
The *in vitro* method developed to determine dialyzable glucose of foods or meals after *in vitro* digestion.

Glucose determination was performed spectrophotometrically using the dinitrosalicylic method (DNS 98%, 12,884-8, Sigma) in a 96-well plate at 562 nm [30]. The method is described briefly in Figure 2.

### 2.5. Data Analysis

Dialyzable glucose, measured at *t* = 0, 30, 60, 90, and 120 min of the second phase of the digestion, was correlated with GI or GL values reported in the literature [24].

Dialyzable glucose ratios (DGR) of meals were correlated with blood glucose concentration ratios (BGR) of meals from the study of Argyri *et al.* [26]. Dialyzable glucose ratios and blood glucose ratios for each time point were calculated as follows (Equations (1) and (2)), using as an example *t* = 120 min.

(1) $$DGR=\frac{dialyzable\;glucose\;120\;min\;after\;the\;initiation\;of\;the\;second\;phase\;of\;the\;in\;vitro\;digestion\;of\;basic\;meal\;\&\;D-dessert}{dialyzable\;glucose\;120\;min\;after\;the\;initiation\;of\;the\;second\;phase\;of\;the\;in\;vitro\;digestion\;of\;basic\;meal}$$

(2) $$BGR=\frac{blood\;glucose\;concentration\;120\;min\;after\;consumption\;of\;basic\;meal\;\&\;D-dessert}{blood\;glucose\;concentration\;120\;min\;after\;consumption\;of\;basic\;meal}$$

All correlations were performed using the non-parametric Spearman’s Rank Correlation Test. *p*-values < 0.05 were considered statistically significant. Statistical analysis was performed with PASW Statistics version 18.0 (SPSS Inc. Chicago, IL, USA).

## 3. Results

The proposed protocol was employed without difficulties for the measurement of dialyzable glucose.

### 3.1. Dialyzable Glucose Released during the in Vitro Digestion of Foods or Meals

**Figure 3:**
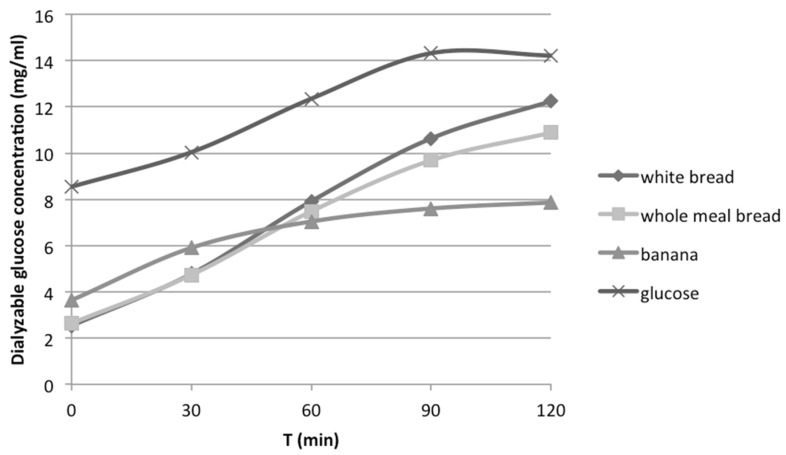
Dialyzable glucose concentration released from carbohydrates during *in vitro* digestion of white bread, whole meal bread, banana and glucose solution.

Dialyzable glucose, measured at *t* = 0, 30, 60, 90 and 120 min after the initiation of the second phase of the *in vitro* digestion, for all the tested foods or meals was plotted in curves. As an example, curves for white bread, whole meal bread, glucose solution and banana are shown in Figure 3.

### 3.2. Correlation of Dialyzable Glucose with GI

**Figure 4:**
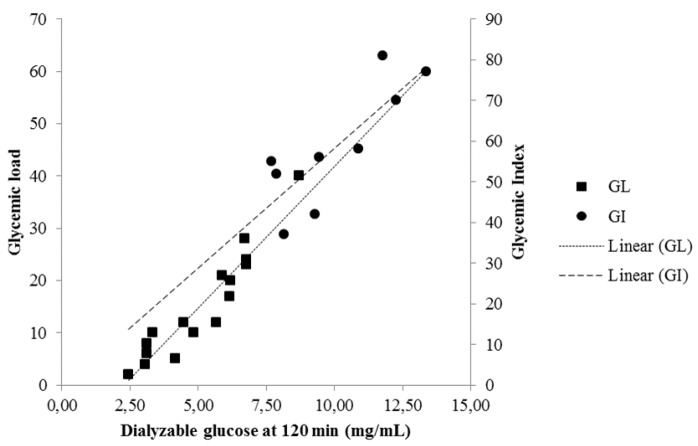
Correlation between dialyzable glucose obtained at 120 min after the initiation of the second phase of the *in vitro* digestion of 16 foods with their published values of GL [24] (Spearman’s rho = 0.953) and nine foods with their published values of GI [24] (Spearman’s rho = 0.800).

Dialyzable glucose measured at *t* = 0, 30, 60, 90 and 120 min after the initiation of the second phase of the *in vitro* digestion for foods in Table 1 was correlated with GI [24] or mean GI when more than one value corresponded to one food (Table 4). The strongest correlation was found when GI was compared with dialyzable glucose measured at 120 min (Spearman’s rho = 0.800, *p* = 0.010) (Figure 4).

Furthermore, a subgroup of high carbohydrate composition foods that included white bread, whole meal bread, white spaghetti, whole meal spaghetti and rice was also correlated with the respective reported values of GI. The correlation coefficient for this subgroup was found to be 1 (*p* < 0.01).

### 3.3. Correlation of Dialyzable Glucose with GL

Dialyzable glucose, measured at *t* = 0, 30, 60, 90 and 120 min after the initiation of the second phase of the *in vitro* digestion for foods in Table 2, was correlated with the respective GL [24] or mean GL when more than one value corresponded to one food (Figure 4). The correlation coefficients are presented in Table 4. A very strong correlation was found between GL and dialyzable glucose at 120 min (Spearman’s rho = 0.953, *p* < 0.001).

### 3.4. Correlation of Dialyzable Glucose with Glycemic Response

**Figure 5:**
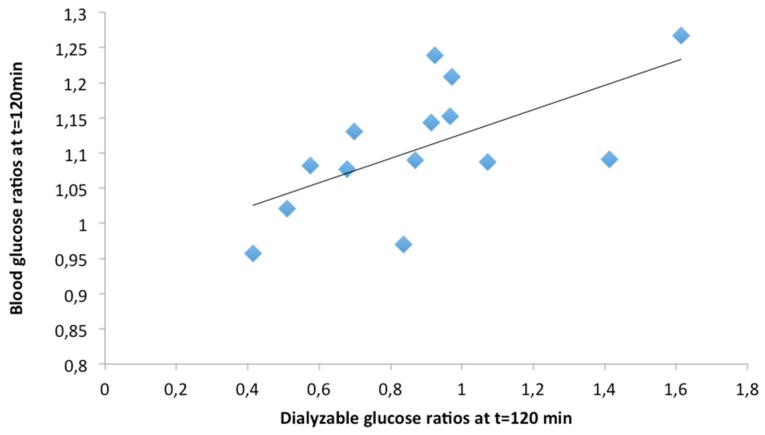
Correlation between dialyzable glucose ratios obtained at 120 min after the initiation of the second phase of the *in vitro* digestion of 14 meals with blood glucose concentration ratios at 120 min after the ingestion of the respective meals by volunteers [26]. Ratios are calculated as dialyzable or blood glucose generated after the *in vitro* digestion or ingestion of each meal to dialyzable or blood glucose generated by the *in vitro* digestion or ingestion of the bread and cheese reference meal. (Spearman’s rho = 0.736).

Dialyzable glucose ratios, estimated from dialyzable glucose obtained during the *in vitro* digestion of the 14 meals (Table 3) at *t* = 0, 30, 60, 90, 120 min after the initiation of the second phase of the digestion process, was correlated with blood glucose ratios in volunteers, at the same time points after the ingestion of the respective meals (Table 4). The highest correlation was found at 120 min (Spearman’s rho = 0.736, *p* = 0.003); dialyzable glucose ratios at 120 min are plotted against blood glucose ratios in Figure 5.

## 4. Discussion

In this study, a novel *in vitro* protocol devised with the aim of predicting glycemic response is described. Two approaches were employed in order to evaluate the proposed method.

The first approach involved the correlation of dialyzable glucose from the *in vitro* digestion of a series of foods with respective GI and GL values as published in the literature [24]. The second approach correlated dialyzable glucose from the *in vitro* digestion of meals with results of glycemic response of the same meals in a clinical trial conducted by our research team [26].

Both approaches revealed strongest correlations of GI or GL or glycemic response with dialyzable glucose in the digests at 120 min after the start of the second phase of the *in vitro* digestion. Therefore, this is the index proposed for the prediction of the glycemic response of a food or meal relative to a control, or to foods or meals of similar composition.

The methodology presented herein reflects on previous studies of other researchers which mimic carbohydrate digestion and subsequently propose an index that reveals glycemic response [12]. In our protocol, novelties in comparison with those studies may be observed as follows:

(a) This *in vitro* digestion method used a dialysis membrane to separate the soluble low molecular weight fraction that reflects the absorbed fraction of glucose or other nutrients [27,29,31,32,33]. Dialysis bags or tubes have been previously employed in *in vitro* methods but in this protocol we propose the use of dialysis membrane fastened with an elastic band to a cylindrical insert in a six-well plate [34]. This approach draws from previous developments in *in vitro* digestion methodology and offers certain practical advantages [35]. In particular, the required amount of food sample is much smaller (2 mL homogenate food) compared to previous proposed methods. This reduced amount of food results in lower amounts and concentrations of reagents and enzymes required. Moreover, the option of stacking six-well plates in the incubator, instead of inserting vials in a space-limiting water bath, facilitates the simultaneous, simple and well-organized testing of many samples. Therefore, it reduces both the time and cost of the analysis and increases efficiency.
(b) The index that reflects glycemic response is dialyzable glucose determined spectrophotometrically at 120 min after the second phase of the *in vitro* digestion. Various indices (carbohydrate digestion rate (rapid/slow), hydrolysis index, glucose equivalents) have been previously used for the correlation of *in vitro* carbohydrate digestion with glycemic response in humans or the GI of meals [13,15,28,36,37,38]. For example, Englyst *et al.* [13] found that the *in vitro* measurement of rapidly available glucose in foods can reflect the glycemic response employing a rapid yet more sophisticated set up than the proposed herein.
(c) The chewing process has been simulated through the use of a homogenizer, followed by treatment with human salivary α-amylase. Simulation of the oral phase is clearly important when carbohydrate digestion is studied. Mechanical breakdown is preferential, as the use of human chewing as employed by other studies raises practical issues when used in routine testing such as inter-subject differences in chewing, enzyme activity, saliva volume as well as other variations between human. These variations limit the ability to achieve reproducible *in vitro* digestion results [15,19]. It must be mentioned, however, that mechanical food breakdown may damage the food matrix thus altering the physical form of the food, a factor that has been argued to affect the glycemic response [4,39,40].
(d) The simultaneous prediction of glycemic response and of mineral bioavailability in one experimental set up may be achieved, a setup which has been already utilized in the prediction of zinc and iron bioavailability. To retain this advantage, the time of pepsin incubation was increased to 120 min as previously proposed [29] although in most *in vitro* carbohydrate digestion protocols this step lasts from 30 to 60 min [12]. It must be noted that other protocols were not initially designed for the prediction of glycemic response [16,17].
(e) The incorporation at the intestinal phase of the digestion process of fat-emulsifying bile salts to aid fat digestion is a comparative advantage of the proposed protocol. Lipid-starch interactions can decrease starch susceptibility to digestion [12] and thus the composition of fat in food has been suggested to effect glycemic response [39].

There are some limitations that must be noted regarding the research presented herein. Primarily, the content of available carbohydrate of foods was not measured but was based on nutrient information from the manufacturer or from food composition tables [25]. This approach has been adopted in similar studies that evaluate *in vitro* protocols for the estimation of glycemic response [16,27,41] or for the prediction of other nutrients [29,42]. Data from different studies were compared, but this allows the inclusion of a larger number of foods in the experimental design [35]. Data on glycemic response of meals employed herein allow the direct comparison with the proposed index, however these values were obtained from diabetic volunteers under oral medication control [26]. An experimental design in the future that includes a large number of foods, analyzed for their carbohydrate content, digested *in vitro* and measured for their GI in healthy subjects in the future would provide a more integrated approach for the proposed protocol. In this respect the study presented herein may be considered as a pilot study that encourages the design of ambitious protocols that will elucidate the applicability of *in vitro* tools.

Rapid *in vitro* methods may not provide numerical values of GI, but an indication/trend of food’s glycemic response [27]. Likewise, the method presented herein allows the comparison of the proposed index to that of a standard or of other foods or meals. Moreover, it appears that the comparison may be more accurate when comparing foods of similar composition as is revealed by comparisons in the subgroup of foods with high carbohydrate composition. Clearly, it was beyond the scope of this protocol to allow single measurements of dialyzable glucose at 120 min to be transformed through a linear regression model to a figure that matches GI or GL. It must be highlighted that *in vitro* approaches may be useful tools but do not always correlate well with *in vivo* values [32] because they cannot precisely imitate human processes. There is no doubt that measuring GI in humans is the best approach. This must be considered when interpreting results obtained with the proposed protocol.

## 5. Conclusions

An *in vitro* methodology that predicts glycemic response was developed and validated. This methodology uses dialyzable glucose at 120 min after the initiation of the second phase of the *in vitro* digestion as a predictor of glycemic response. The proposed protocol is rapid, simple, of relatively low cost, and requires minimal skills and infrastructure whilst allowing the simultaneous testing of multiple samples. It offers a useful tool in the screening of several foods or meals before selecting a limited number for the further measurement of glycemic response in humans.

## Figures and Tables

**Table 1:** Nutrient content of foods digested *in vitro* for the correlation of dialyzable glucose with published glycemic index (GI). Each food contained 0.25 g available carbohydrate ^1^.

Food Item	Quantity (g)	Sugar (g)	Fiber (g)	Fat (g)	Protein (g)
**Breakfast cereals**					
Chocolate toasted rice ^i^	0.290	0.102	0.006	0.007	0.015
Corn flakes ^ii^	0.300	0.024	0.009	0.003	0.021
Whole wheat flakes and rolled raisins and roasted sliced hazelnuts and almonds ^iii^	0.390	0.064	0.030	0.024	0.043
**Cereal grains**					
Rice long-grain ^iv^	0.870	0.002	0.003	0.004	0.024
Spaghetti n.5 white ^v^	0.780	0.012	0.011	0.005	0.044
Spaghetti n.5 whole meal ^vi^	0.850	0.015	0.022	0.011	0.055
**Fruit**					
Banana ^vii^	1.250	nr *	0.030	0.011	0.011
**Breads**					
White bread ^vii^	0.510	0.033	0.013	0.025	0.038
Whole meal bread ^ix^	0.570	0.036	0.030	0.029	0.046

* “nr”: non reported value; ^1^ nutrient contents according to the manufacturer or food composition database; ^i^ Coco Pops, Kellogg’s, Spain, 165; ^ii^ Corn Flakes The Original, Kellogg’s, Spain, 168; ^iii^ Alpen Muesli—No added Sugar, Elgeka, Weetabix, England, 198; ^iv^ Uncle Ben’s, Mars Hellas, 277; ^v^ Barilla, Barilla G.eR. Fratelli, Italy, 533; ^vi^ Barilla, Barilla G.eR. Fratelli, Italy, 537; ^vii^ imported banana, South America, 397; ^vii^ Mcennedy, Lidl Stiftung & Co, Germany, 101; ^ix^ Mcennedy, Lidl Stiftung & Co, Germany, 85.

**Table 2:** Nutrient content of foods digested *in vitro* for the correlation of dialyzable glucose with glycemic load (GL) ^i^.

Food Item	Quantity	Available Carbohydrates (g)	Sugar (g)	Fiber (g)	Fat (g)	Protein (g)
**Beverages**						
Energy drink with orange flavor ^1^	2.5 mL	0.350	0.350	0.000	0.000	0.000
Carbonated orange juice ^2^	2.5 mL	0.318	0.318	0.000	0.000	0.000
Natural apple juice ^3^	2.5 mL	0.268	0.258	0.030	0.000	0.003
**Breakfast cereals**						
Chocolate toasted rice ^4^	0.3 g	0.255	0.105	0.006	0.008	0.015
Corn flakes ^5^	0.3 g	0.252	0.024	0.009	0.003	0.021
Whole wheat flakes and rolled raisins and roasted sliced hazelnuts and almonds ^6^	0.3 g	0.190	0.049	0.023	0.019	0.033
**Cereal grains**						
Rice long-grain ^7^	1.5 g	0.430	0.008	0.015	0.020	0.110
Spaghetti n.5 white ^8^	1.5 g	0.480	0.053	0.045	0.023	0.188
Spaghetti n.5 whole meal ^9^	1.5 g	0.400	0.053	0.077	0.038	0.188
**Fruit**						
Apple ^10^	1.2 g	0.160	nr *	0.032	tr **	tr **
Banana ^11^	1.2 g	0.240	nr *	0.029	0.010	0.010
**Breads**						
White bread ^12^	0.3 g	0.147	0.020	0.008	0.014	0.023
Whole meal bread ^13^	0.3 g	0.132	0.019	0.016	0.015	0.024
**Legumes**						
Small lentils ^14^	1.5 g	0.180	nr *	0.118	0.008	0.136
**Dairy products**						
Skim milk ^15^	2.5 g	0.119	nr *	nr *	0.000	0.085
**Infant formula**						
Milk for infant ^16^	1.0 g	0.580	nr *	nr *	0.280	0.090

* “nr”: non reported value; ** “tr”: traces; ^i^ nutrient contents according to the manufacturer or food composition database; ^1^ Lucozade, GlaxoSmithKline, UK, 24; ^2^ Fanta, Coca Cola 3E, Greece, 23; ^3^ Amita, Coca Cola 3E, Greece, 32; ^4^ Coco Pops, Kellogg’s, Spain, 165; ^5^ Corn Flakes The Original, Kellogg’s, Spain, 168; ^6^ Alpen Muesli—No added Sugar, Elgeka, Weetabix, England, 198; ^7^ Uncle Ben’s, Mars Hellas, 277; ^8^ Barilla, Barilla G.eR. Fratelli, Italy, 533; ^9^ Barilla, Barilla G.eR. Fratelli, Italy, 537; ^10^ Golden variety, Greece, 388; ^11^ imported banana, South America, 397; ^12^ Mcennedy, Lidl Stiftung & Co, Germany, 101; ^13^ Mcennedy, Lidl Stiftung & Co, Germany, 85; ^14^ small lentils imported from Canada, Sklavenitis, Greece, 462; ^15^ Mevgal, Greece, 373; ^16^ Nan-1 milk infant formula, Nestlé Hellas, 445.

**Table 3:** Nutrient content of basic meal and desserts digested *in vitro* and ingested by volunteers (g/portion) [26]. The portion is shown in parenthesis beside each product *.

Tested Foods	Fat (g)	Carbohydrates (g)	Sugar (g)	Fiber (g)	Protein (g)
White bread (24 g) ^1^	1.2	14.900	0.800	1.300	2.500
Cheese (20 g) ^2^	1.7	0.300	nr *	0.000	5.600
C-chocolate (30 g) ^3^	9.0	11.900	10.900	2.000	1.300
D-chocolate (30 g) ^4^	5.3	7.300	0.100	2.700	0.800
C-jelly strawberry (165 g) ^3^	0.0	27.100	26.700	0.300	2.700
D-jelly strawberry (165 g) ^4^	0.0	8.400	0.100	2.600	3.400
C-milk dessert (160 g) ^3^	7.3	32.300	25.900	0.000	5.300
D-milk dessert (160 g) ^4^	2.3	22.900	7.400	5.100	5.200
C-crème caramel (120 g) ^3^	4.6	24.400	24.400	0.200	3.800
D-crème caramel (120 g) ^4^	1.2	7.800	0.300	4.000	0.900
C-cake (55 g) ^3^	8.7	32.500	18.400	0.400	3.600
D-cake (55 g) ^4^	8.0	20.000	0.200	3.700	3.400
C-mille-feuille (90 g) ^3^	11.2	28.100	15.500	0.800	3.100
D-mille-feuille (90 g) ^4^	4.0	17.900	0.200	3.700	2.500
C-pastry cream (65 g) ^3^	3.2	15.700	12.700	0.100	1.700
D-pastry cream (65 g) ^4^	1.5	9.900	0.240	2.700	1.600

* nr: non reported value; ** nutrient contents according to the manufacturer or food composition database; ^1^ Kris-Kris, Elbisco, Greece; ^2^ Fina, Milko Sverige, Sweden; ^3^ C-dessert, Jotis SA, Greece; ^4^ D-dessert, Jotis SA, Greece.

**Table 4:** Spearman’s correlation between dialyzable glucose obtained at *t* = 0, 30, 60, 90, and 120 min after the initiation of the second phase of the *in vitro* digestion process of: (a) 16 foods with their published values of GL [24]; (b) nine foods with their published values of GI [24]; and (c) 14 meals with blood glucose concentration at *t* = 0, 30, 60, 90, and 120 min after the ingestion of the respective meals by volunteers [26].

	Dialyzable Glucose *vs.* GL		Dialyzable Glucose *vs.* GI		Dialyzable Glucose Ratios ^1^ *vs.* Blood Glucose Ratios ^1^	
Time (min)	Spearman’s rho	*p*	Spearman’s rho	*p*	Spearman’s rho	*p*
0	0.656	0.006	0.333	0.381	0.152	0.605
30	0.723	0.002	0.500	0.170	0.490	0.075
60	0.833	<0.001	0.667	0.050	0.363	0.203
90	0.854	<0.001	0.750	0.020	0.336	0.240
120	0.953	<0.001	0.800	0.010	0.736	0.003

^1^ Ratios are calculated as dialyzable or blood glucose generated after the *in vitro* digestion or ingestion of each of the 14 meals to dialyzable or blood glucose generated by the *in vitro* digestion or ingestion of the bread and cheese reference meal.
